# High-Throughput Metabolomic Profiling of Skin Lesions: Comparative Study of Cutaneous Squamous Cell Carcinoma, Basal Cell Carcinoma, and Normal Skin Via e-Biopsy Sampling

**DOI:** 10.1007/s12195-025-00846-1

**Published:** 2025-04-03

**Authors:** Leetal Louie, Julia Wise, Ariel Berl, Ofir Shir-az, Vladimir Kravtsov, Zohar Yakhini, Avshalom Shalom, Alexander Golberg, Edward Vitkin

**Affiliations:** 1 School of Mechanical Engineering, Tel Aviv University, Tel Aviv, Israel; 2 Arazi School of Computer Science, Reichman University, Herzliya, Israel; 3 Department of Plastic Surgery, Meir Medical Center, Kfar Sava, Israel; 4 Department of Pathology, Meir Medical Center, Kfar Sava, Israel; 5 Department of Computer Science, Technion - Israel Institute of Technology, Haifa, Israel

**Keywords:** High-throughput metabolomics, Metabolomic profiles, Cutaneous squamous cell carcinoma, Basal cell carcinoma, Electroporation-based biopsy, E-Biopsy

## Abstract

**Purpose:**

Rising rates of cutaneous squamous cell carcinoma (cSCC) and basal cell carcinoma (BCC) make standard histopathology diagnostic methods a bottleneck. Using tissue molecular information for diagnostics offers a promising alternative. Faster specimen collection and high-throughput molecular identification can improve the processing of the increasing number of tumors. This study aims (i) to confirm the ability of e-biopsy technique to harvest metabolites, (ii) to obtain high-resolution metabolomic profiles of cSCC, BCC, and healthy skin tissues, and (iii) to perform a comparative analysis of the collected profiles.

**Methods:**

Tumor specimens were collected with electroporation-based biopsy (e-biopsy), a minimally invasive sampling collection tool, from 13 tissue samples (cSCC, BCC, and healthy skin) from 12 patients. Ultra performance liquid chromatography and tandem mass spectrometry (UPLC-MS-MS) was used for molecular identification and quantification of resulting metabolomic profiles.

**Results:**

Here we report measurements of 2325 small metabolites identified (301 with high confidence) in 13 tissue samples from 12 patients. Comparative analysis identified 34 significantly (p < 0.05) differentially expressed high-confidence metabolites. Generally, we observed a greater number of metabolites with higher expression, in cSCC and in BCC compared to healthy tissues, belonging to the subclass amino acids, peptides, and analogues.

**Conclusions:**

These findings confirm the ability of e-biopsy technique to obtain high-resolution metabolomic profiles suitable to downstream bioinformatics analysis. This highlights the potential of e-biopsy coupled with UPLC-MS-MS for rapid, high-throughput metabolomic profiling in skin cancers and supports its utility as a promising diagnostic alternative to standard histopathology.

**Supplementary Information:**

The online version contains supplementary material available at 10.1007/s12195-025-00846-1.

## Introduction

There is an observed rise in incidence rates of cutaneous squamous cell carcinoma (cSCC) and basal cell carcinoma (BCC) [1–5]. These cancers are commonly diagnosed but often not included in cancer registries due to their low mortality rate [4], despite their impact on quality of life and the risk of premature mortality [2, 6]. These trends bring attention to a potential increase in diagnostic wait times using the current gold standard method of tissue excision followed by histopathological examination [7]. Excision may also be problematic when subsequent treatment requires electrodesiccation and cautery [8]. In consideration of this, it is important to explore less invasive and time-efficient methods for distinguishing between healthy and cancerous skin, as well as between different types of skin cancers. Faster turnaround times would be beneficial considering the varying aggressiveness and likelihood of metastasis of cSCC compared to BCC [9].

The potential of molecular profiling in diagnostics is evident given the observed capability to collect samples of molecular information from cSCC and BCC lesioned skin with simpler and faster methods than the current gold standard. To address the need for biomarker sampling we developed a novel tissue sampling approach with molecular biopsy using electroporation. Electroporation-based technologies have been successfully used to permeabilize the cell membrane *in vivo*, enabling a wide set of applications ranging from tumor ablation to targeted delivery of molecules to cell populations and tissues [10]. More recently, we have shown that electroporation-based molecular sampling, termed “e-biopsy”, selectively extracts liquids from solid tissues with informative proteomes in animal models in liver cancer [11] and brain melanoma [12] *in vitro* and from breast cancer *in vivo*, enabling in vivo spatial mapping of differential protein expression [13]. The analysis of molecular information obtained from specimens collected with this method has successfully differentiated between healthy and cancerous human skin tissues [14–16]. Combined with high-throughput analytics that have demonstrated their ability to identify genes, proteins, lipids, and metabolites of cSCC and BCC [17–25], e-biopsy is a promising direction for analysis of water-soluble molecules of skin cancers. E-biopsy broadens the spectrum of capabilities of handheld devices in cancer diagnostics and differs from tools such as the iKnife [26, 27] and MasSpec Pen [28, 29] which have been designed for intraoperative use and require real-time connection to a mass spectrometer. However, the sampling of small metabolites with e-biopsy has not been reported.

Metabolomic analyses of cSCC and BCC have previously reported differences in cancerous tissues compared to healthy [18–20, 22, 23]. These reports have used a variety of analysis methods such as high-resolution magic angle spinning (HR-MAS) 1H nuclear magnetic resonance (NMR) spectroscopy [18], liquid chromatography tandem mass spectrometry (LC-MS-MS) [19], triple-quadrupole MS (QqQMS) [20], ultraperformance LC coupled to a time-of-flight tandem MS (UPLC-TOF-MS/MS) [22], and 1H NMR spectroscopy [23], and have identified 9, 27, 27, 181, and 8 significant metabolites, respectively. These studies used both tissue [18, 19, 22, 23] and serum [20] samples. Lacking here are promising minimally invasive specimen collection methods that can provide inputs into these high-throughput analyses for metabolite identification.

Here we present the first comparative analysis of high-throughput metabolomic profiling of cSCC, BCC, and healthy skin tissues sampled with the e-biopsy method. This study contributes to the recently discovered capabilities of e-biopsy in transcriptomics, proteomics, and lipidomics [14–17, 24, 25].

## Materials and Methods

### Human Patients

Table 1 contains patient sex, age, and tumor type. This study was approved by the Meir Medical Center IRB, number MMC-19–0230. All patients gave written consent for participation and performance of molecular analysis of their sample tissue.

**Table 1.** Patient sex, age, and tumor type

Patient	Sex	Age	Tumor type
1	Male	73	SCC
2	Female	85	SCC
3	Male	56	SCC
4	Male	85	SCC
5	Female	69	BCC
6	Male	71	BCC
7	Female	91	BCC
8	Female	74	BCC
9	Male	81	BCC
10	Female	91	BCC
11	Female	74	Healthy
12	Female	57	Healthy

### Sample Collection

**Fig. 1.**
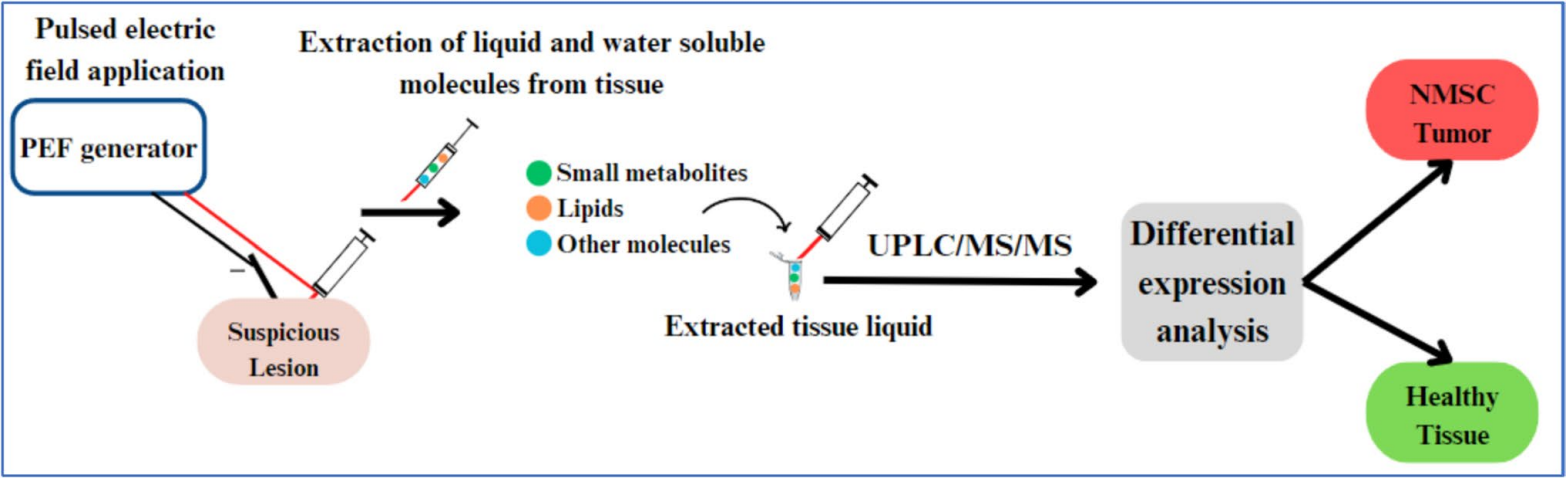
Study workflow: A pulsed electric field (PEF) is delivered to a tissue sample for extraction of water-soluble molecules and sample extracts are stored in 1.5 mL tubes until ready for UPLC-MS-MS analysis. Data from UPLC-MS-MS is used for differential expression analysis

Ten tissue samples were collected from 10 patients undergoing surgical excision of cSCC or BCC between March 2020 and March 2022 at Meir Medical Center, Israel. Additionally, 3 healthy tissue samples were collected from 2 patients undergoing blepharoplasty. The diameter of excised tissue was at least 1 cm. Between 10–20 minutes after surgery, a total of 13 fresh tissue samples underwent e-biopsy extraction methods. Following this, UPLC-MS-MS and differential expression analyses were performed. The workflow of the e-biopsy and subsequent analysis are summarized in Fig. 1**.**

A standard 30-G insulin syringe with a needle is inserted in the sampling location and the ground electrode (custom made with a 3 mm diameter) is positioned approximately 5 mm apart on the skin surface, without penetration. A pulsed electric field (PEF) is delivered through the sampling needle and then a vacuum is applied to the needle to drive the released cellular content into it and the syringe. PEF settings are a combination of high-voltage short pulses and low-voltage long pulses [14, 30]: 40 pulses, 1000 V, 40 µs, 4 Hz, and 40 pulses, 50 V, 5 ms, 4 Hz. The syringe used to collect liquids from tissues is 1.5 ml and the vacuum on the needle is applied manually. Immediately following, liquids are transferred to 1.5 ml tubes that contained 100 µl double distilled water and placed in − 20℃. A custom-made high-voltage pulsed electric field generator is used for PEF application [31].

Samples were stored until shipped to Beijing Genomics Institute for analysis.

### UPLC-MS-MS Analysis

UPLC-MS-MS analysis was performed by Beijing Genomics Institute. An ACQUITY UPLC BEH C18 column and UPLC BEH Amide column (both 1.7 µm, 2.1 × 100 mm, Waters, USA) in both positive and negative modes were used for chromatographic separation (4 analyses in total). Water 2D UPLC (Waters, USA) and tandem Q Exactive high resolution mass spectrometer (Thermo Fisher Scientific, USA) with a heated electrospray ionization (HESI) source were used for this analysis. Xcalibur 2.3 software was used. Data gathered included metabolite ID, ID reliability level (graded levels 1 to 4, with 1 and 2 being the most accurately identified metabolites used for subsequent differential screening), and metabolite intensity (Tables S1–S4). This data was used for the analysis of differential metabolite abundance. Detailed UPLC-MS-MS information and methods can be found in the Supplementary methods.

### Power Analysis

Power analysis was performed separately for each tissue type using *TTestIndPower.solve_power()* function from *statsmodels.stats* Python library. The effect size was calculated as the average absolute Cohen’s d for each reliable metabolite (graded as level 1 or level 2), resulting in effect sizes of 0.67, 0.53, and 0.76 for cSCC, BCC, and Healthy conditions, respectively. The corresponding observed experimental power was 17.7, 14.0 and 18.5% for cSCC, BCC, and Healthy conditions, respectively.

### Differential Metabolite Screening

**Fig. 2.**
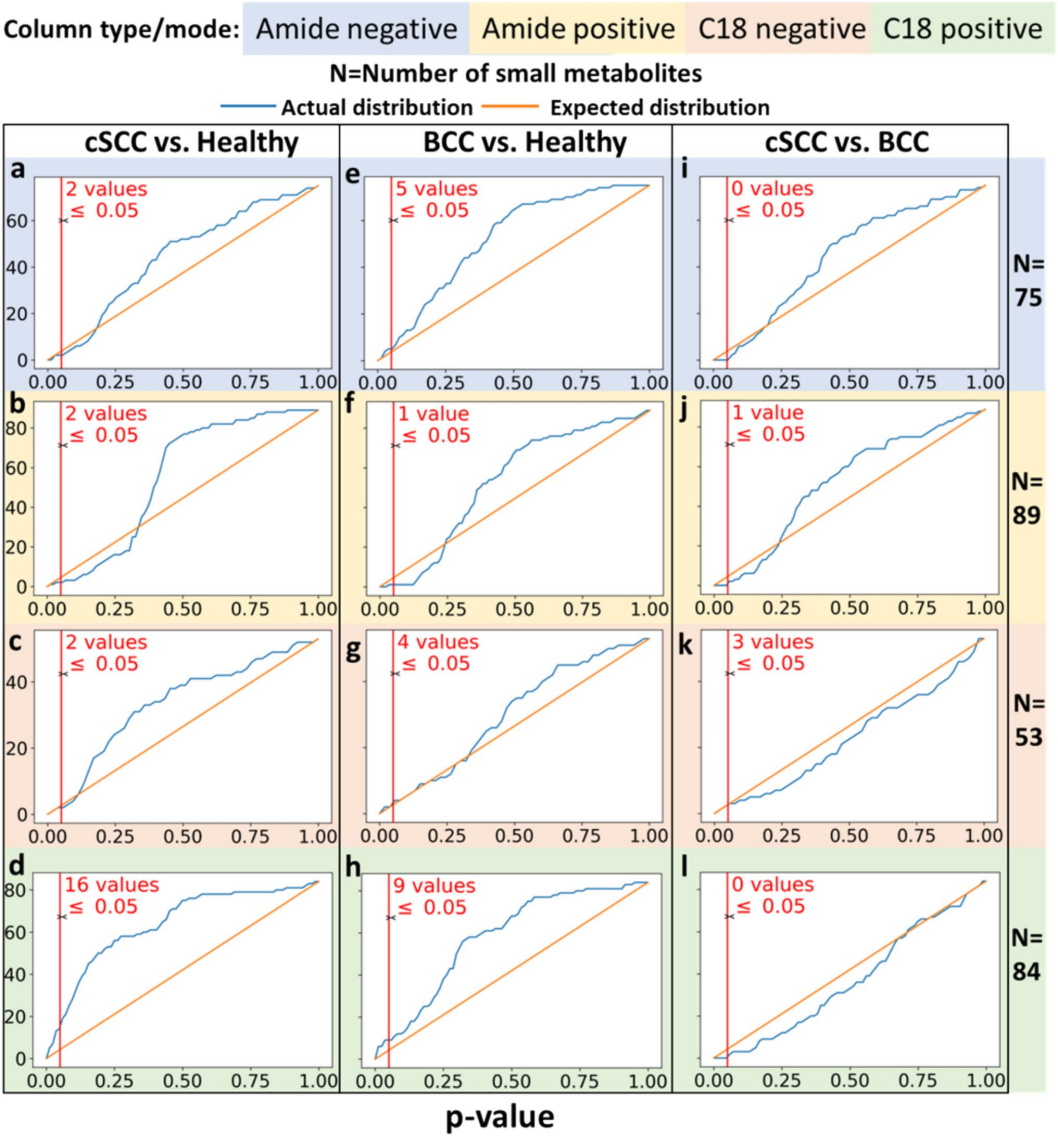
**a**–**l** Overabundance plots comparing the distribution of metabolites differential expression (both over- and under-expression) p-values between control (normal skin tissue), cSCC, BCC tumor samples. Results from 2 chromatography column types and mode is shown by the background color. A total of 13 samples, and 301 metabolites extracted by e-biopsy were analyzed. **a**–**d** cSCC vs. Healthy, **e**–**h** BCC vs. healthy and **i**–**l** cSCC vs. BCC

**Fig. 3.**
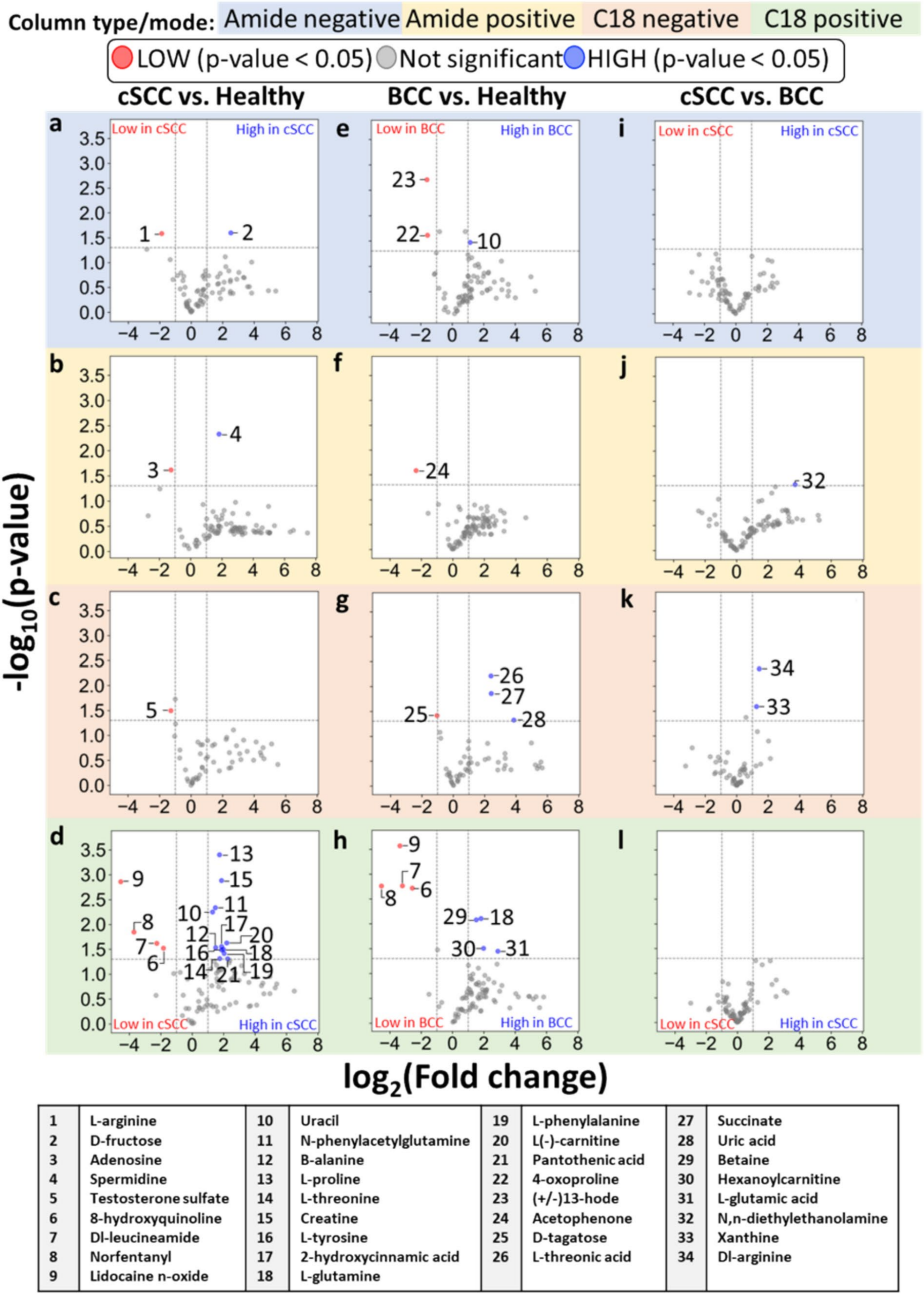
**a**–**l** Volcano plots showing the fold-change difference of metabolite intensities. **a**–**d** cSCC vs. Healthy. **b** BCC vs. Healthy. **c** cSCC vs. BCC. Numbered datapoints correspond to metabolite names found in the table below the plots. Fold-change and p-value data for the metabolites identified in the table can be found in Tables 2, S5–S7. High-resolution plots can be found in Figs. S1–S12

Differential metabolite screening was performed on all metabolite intensities measured by UPLC-MS-MS (Tables S1–S4). Student’s t-test and fold change analysis between average metabolite intensities were performed for each metabolite in each comparison pair of groups (cSCC vs. healthy, BCC vs. healthy, and cSCC vs. BCC). Resulting p-values and fold change values were used for overabundance analysis and volcano analysis (Figs. 2, 3).

#### Statistical Overabundance Analysis

Overabundance analysis verifies that compounds have different abundance levels when comparing two classes of samples by comparing the actual and expected distribution of p-values [32]. This analysis is used in analyses with multiple comparisons and explores internal data variability. The analysis relies only on the number of compounds (i.e., metabolites) and their observed p-values (obtained here from the Student’s t-test). p-value correction was calculated with the Benjamini-Hochberg approach. FDR was calculated as the ratio of expected and observed values below an uncorrected p-value of interest. The distribution of the expected p-values was generated from a null model assuming the same number of compounds (Fig. 2).

#### Fold Change and Volcano Plot Analysis

A volcano plot depicts the magnitude of fold change of metabolites and differential significance between two analyzed populations. Differentially expressed metabolites were defined for this analysis as those with a − *log*10(*p*-value) > 1.3 (i.e., unpaired Student t-test *p*-value < 0.05) and those with *− *1* < log*2(*fold-change*) < 1. Fold change was calculated using the average of intensity values for each comparison group i.e., *fold-change*(*metabolite*) = *avg*(*grp1*)/*avg*(*grp2*). The data was then filtered to include only the compounds with high reliability score (levels 1 and 2) (Fig. 3**)**. The data of the most interesting compounds were organized into tables to showcase their associated p-values and fold change values (Tables 2, S5–S7).

**Table 2.** Differentially expressed metabolites with associated fold change values and p-values

cSCC vs. Healthy Fold-change (p-value)	BCC vs. Healthy Fold-change (p-value)	cSCC vs. BCC Fold-change (p-value)
**L-glutamine** 3.97 (0.03)	**L-glutamine** 3.54 (0.008)	**DL-arginine** 2.71 (0.005)
**Dl-leucineamide** 0.21 (0.02)	**Dl-leucineamide** 0.11 (0.002)	**Xanthine** 2.40 (0.03)
**Norfentanyl** 0.08 (0.01)	**Norfentanyl** 0.04 (0.002)	**N,N-diethylethanolamine** 13.15 (0.05)
**Lidocaine n-oxide** 0.04 (0.001)	**Lidocaine n-oxide** 0.1 (0.0003)	
**8-hydroxyquinoline** 0.28 (0.03)	**8-hydroxyquinoline** 0.17 (0.002)	
**Uracil** 2.46 (0.006)	**Uracil** 2.20 (0.03)	
**Testosterone sulfate** 0.41(0.03)	**Succinate** 5.38 (0.01)	
**L(-)-carnitine** 4.60 (0.02)	**L-threonic acid** 5.31 (0.006)	
**Β-alanine** 2.83 (0.03)	**D-tagatose** 0.49 (0.04)	
**L-threonine** 3.37 (0.05)	**Uric acid** 14.58 (0.05)	
**Creatine** 3.66 (0.001)	**L-glutamic acid** 7.46 (0.04)	
**L-proline** 3.34 (0.0004)	**Betaine** 2.87 (0.008)	
**2-hydroxycinnamic acid** 3.67 (0.03)	**Hexanoylcarnitine** 3.96 (0.03)	
**L-tyrosine** 3.66 (0.03)	**(+/-)13-hode** 0.33 (0.002)	
**L-phenylalanine** 4.11 (0.04)	**4-oxoproline** 0.34 (0.02)	
**Pantothenic acid** 4.79 (0.05)	**Acetophenone** 0.2 (0.03)	
**N-phenylacetylglutamine** 2.79 (0.005)		
**L-arginine** 0.27 (0.03)		
**D-fructose** 5.75 (0.03)		
**Spermidine** 3.54 (0.005)		
**Adenosine** 0.42 (0.02)		

Columns contain the metabolites (in bold) identified as significant by the pairwise comparison groups cSCC vs. Healthy, BCC vs. Healthy and cSCC vs. BCC. An additional column is added for metabolites found in both cSCC and BCC compared to Healthy. *Reading example*: Dl-arginine ratio of its average intensity in cSCC tissues to average intensity in BCC tissues is 2.71. This comparison has a Student t-test p-value of 0.005

## Results

A total of 2325 metabolites were identified across 2 different columns in 2 modes (*Methods*) and then filtered to 301 eligible for differential expression according to the measurement reliability level, omitting metabolites at level 3 and 4. Eligible metabolites were used for the following groupwise comparisons: cSCC vs. Healthy, BCC vs. Healthy, and cSCC vs. BCC. Overabundance plots (Fig. 2) present analysis results, and the number of metabolites with Student t-test p-value below 0.05 is highlighted in red. Volcano plots (Fig. 3) show relative group affinity of each metabolite, highlighting significantly over- and under-expressed metabolites. The major findings are summarized in Table 2.

### Metabolomic Changes Between Squamous Cell Carcinoma and Healthy Samples

Across all column types and modes, the overabundance analysis of cSCC compared to healthy skin revealed a total of 6 metabolites with Student t-test p-values below 0.01 (FDR = 0.5) and 22 with p-values below 0.05 (including 3 after correction, FDR = 0.68, Fig. 2a–d), with 14 significantly (fold change > 2) higher in cSCC and 7 significantly lower in cSCC (Fig. 3a–d**).** The amide column in negative mode (Fig. 3a) identified L-arginine at lower intensity and D-fructose at higher intensity in cSCC. The amide column in positive mode (Fig. 3b) identified adenosine at lower intensity and spermidine at higher intensity in cSCC. The C18 column in negative mode (Fig. 3c) identified testosterone sulfate at lower intensity in cSCC. The C18 column in positive mode (Fig. 3d) identified 8-hydroxyquinoline, DI-leucineamide, norfentanyl, and lidocaine n-oxide at lower intensity, and uracil, n-phenylacetylglutamine, B-alanine, L-proline, L-threonine, L-tyrosine, 2-hydroxycinnamic acid, L-glutamine, L-phenylalanine, L(-)-carnitine, pantothenic acid and creatine at higher intensity in cSCC. Moreover, l-proline was identified with p-value = 4e−4 (corrected p-value 0.03). High-resolution volcano plot of this comparison can be viewed in the supplementary material (Figs. S1–S4). All associated p-values and fold change values for the metabolites listed above can be found in Tables 2 and S5.

### Metabolomic Changes Between Basal Cell Carcinoma and Healthy Samples BCC vs. Healthy

Across all column types and modes, the overabundance analysis of BCC compared to healthy skin revealed a total of 8 metabolites with Student t-test p-values below 0.01 (FDR = 0.38) and 19 with p-values below 0.05 (FDR = 0.79), including 4 after Benjamini-Hochberg correction (Fig. 2e–h), with 8 significantly (fold change > 2) higher in BCC and 8 significantly lower in cSCC (Fig. 3e–h**).** The amide column in negative mode (Fig. 3e) identified 4-oxoproline and (+/−)13-hode at lower intensity and uracil at higher intensity in BCC. The amide column in positive mode (Fig. 3f) identified acetophenone at lower intensity in BCC. The C18 column in negative mode (Fig. 3g) identified D-tagatose at lower intensity and L-threonic acid, succinate, and uric acid at higher intensity in BCC. The C18 column in positive mode (Fig. 3h) identified 8-hydroxyquinoline, DI-leucineamide, norfentanyl, and lidocaine n-oxide at lower intensity, and L-glutamine, betaine, hexanoylcarnitine, and L-glutamic acid at higher intensity in BCC. Moreover, Dl-leucineamide was identified with p-value = 2e−3 (corrected p-value 0.04). High-resolution volcano plot of this comparison can be viewed in the supplementary material (Figs. S5–S8). All associated p and fold change values for the metabolites listed above can be found in Tables 2 and S6.

### Comparative Analysis of Metabolomic Profiles of Basal and Squamous Cell Carcinoma Samples

Across all column types and modes, the overabundance analysis of cSCC compared to BCC revealed 1 metabolite (DL-arginine) with Student t-test p-values below 0.01 and 4 metabolites with p-values below 0.05 (FDR > 1, Fig. 2i–l), with 3 significantly (fold change > 2) higher in cSCC and none significantly lower (Fig. 3i–l**).** The amide column in negative mode (Fig. 3i) did not identify differentially expressed metabolites. The amide column in positive mode (Fig. 3j) identified N,N-diethylethanolamine at higher intensity in cSCC. The C18 column in negative mode (Fig. 3k) identified xanthine and DL-arginine at higher intensity in cSCC. The C18 column in positive mode (Fig. 3l) did not identify differentially expressed metabolites. High-resolution volcano plot of this comparison can be viewed in the supplementary material (Figs. S9–S12). All associated p and fold change values for the metabolites listed above can be found in Tables 2 and S7.

## Discussion

A comparative high-throughput analysis of metabolomic profiles was performed on cSCC, BCC, and healthy skin tissues sampled with e-biopsy technique. The results suggest a difference in metabolomic profiles between cancer and healthy tissues. The most notable trends in the data are the abundance of metabolites in the subclass of amino acids, peptides, and analogues. Of the total 34 identified differentially expressed metabolites, 14 fall into this subclass. Of the 14 in this subclass, 10 were found to be present in higher quantities in the cancerous tissues compared to healthy tissues and 1 higher in cSCC compared to BCC. Additionally, the 2 significant metabolites of the subclass carbohydrates and carbohydrate conjugates were both found to be higher in the cancerous tissues compared to healthy. There do not appear to be comparison-group wide trends for the remaining significant compounds.

To the best of our knowledge, no previous metabolomic profiling studies using e-biopsy molecular harvesting from cSCC and BCC were performed. Previous studies comparing cSCC to healthy skin have used QqQMS [20], UPLC-TOF-MS/MS and nLC-MS/MS [22], and 1H NMR spectroscopy [23]. Previous studies comparing BCC to healthy skin tissue have used high-resolution magic angle spinning 1H NMR [18] and LC-MS-MS [19]. An advantage of the e-biopsy method is that sample preparation does not require overnight storage as with some of these previously noted methods [20, 22, 23].

Our study contributes to the growing body of knowledge of molecular markers of cSCC and BCC, helping to pave the way for molecular diagnostics with potential to outperform the current methods. E-biopsy coupled with UPLC-MS-MS was able to reliably identify 301 different metabolites, with similarities to previous metabolomic studies of cSCC and BCC. As with our study, a previous study found phenylalanine, fructose and tyrosine increased in cSCC versus healthy [20]. This study also found higher levels of glutamic acid in cSCC versus healthy [20], however we found glutamic acid differentially expressed in BCC versus healthy samples only. As in our study, there are previous reports of increased uracil in BCC versus healthy samples [33], however, we also found higher levels of uracil in cSCC versus healthy. Pantothenic acid and betaine were previously reported to be lower in BCC compared to healthy skin [19], whereas we found higher betaine in BCC and higher pantothenic acid in cSCC. Unlike previous research that identified lower creatine in BCC compared to healthy skin [18], we observed higher creatine in comparison of cSCC to healthy skin tissue. A previous study of cSCC compared to healthy skin found higher levels of creatine, tyrosine, and glutamine [23], which is consistent with our results. Again, consistent with our results, another comparison of cSCC to healthy found threonine, creatine, proline, phenylalanine, pantothenic acid, spermidine, uracil, and glutamine higher in cSCC compared to healthy [22]. This study also found higher adenosine present in cSCC compared to healthy skin [22], whereas we observed it in lower levels in cSCC. The same study found that arginine was higher in cSCC compared to healthy [22], but our study observed arginine lower in cSCC compared to healthy though higher in cSCC compared to BCC. The difference in the arginine measurements may result from different enantiomers, but this is not clear from the data previously reported [22]. Other metabolites identified by UPLC-TOF-MS/MS and nLC-MS/MS that were also identified here are glutamic acid, betaine, and hexanoylcarnitine [22]. These 3 metabolites however, were not identified as significant in the comparison of cSCC to healthy, rather they were significant in BCC compared to healthy. Lastly, xanthine was previously found significantly higher in cSCC compared to healthy skin [22], whereas we found it higher in cSCC compared to BCC. The observed discrepancies could be mainly attributed both to analyzed tissues as well as to selected healthy controls. Previous studies analyzed serum samples or skin tissues collected from varied locations such as eyelids, chest, face, and penis. Additionally, sources of healthy controls varied. Serum was collected from a healthy population, healthy skin was taken beyond tumor margins at different distances from the cancerous tissue or from patients with different cancers undergoing repair surgery. Finally, each study used a different extraction technique and a data analysis method. Still, we believe that the similarities observed with previous studies demonstrate that e-biopsy approach is a comparable tool for metabolomic profiling of tissues.

Several potentially interesting differentially expressed metabolites were identified in our study. Cinnamic acid, whose derivative is hydroxycinnamic acid, has been observed to reduce proliferation of cells in glioblastoma, melanoma, prostate, and lung cancers [34]. Additionally, cinnamic acid and its derivatives have been shown to trigger apoptosis in melanoma cells [35]. As with 2-hydroxycinnamic acid, phenylalanine and tyrosine which are precursors to cinnamic acid [36] were also found in higher quantities in cSCC samples. Phenylalanine and tyrosine have also both been examined as biomarkers of skin cancer [37]. B-alanine, found higher in cSCC, is involved in mechanisms of itch and pain [38] which are symptoms associated with cSCC [39]. Creatine, which we found at higher levels in cSCC, is created in the generation of ATP [40] that provides energy for cells. SCC and BCC have been found to have higher total activity of certain creatine kinase isozymes compared to normal human skin [40]. L-proline, which we found higher in cSCC, was reported to suppress actinic skin cell damage after solar stimulated UV radiation [41], possibly suggesting that it is produced in abundance in cSCC as a defense mechanism. Follow-up studies with in-depth analysis of specific metabolites and a larger patient cohort should be performed for validation of these findings.

One of the key limitations of this study is the representativeness of the control group used for comparisons. Tissue samples from patients undergoing blepharoplasty, while convenient, do not fully capture the heterogeneity inherent in non-cancerous skin conditions. The skin from periocular regions in these patients may differ significantly in molecular composition, structure, and environmental exposure compared to healthy skin from other body sites or from individuals without surgical indications. As such, these samples may not provide an ideal baseline for distinguishing malignant lesions from truly representative non-cancerous skin. To address this limitation, future studies should incorporate a more diverse population of healthy tissue samples from individuals with varying demographics, anatomical locations, and environmental exposures. This would ensure a more comprehensive understanding of the molecular differences between cancerous and non-cancerous skin. Limitations of this study include a sample size that is not representative enough for drawing conclusions about metabolite behavior. Power analysis (*Methods*) based on the observed effect size revealed that the available sample size provides only ~ 15% statistical power for detecting meaningful differences in our study. Comparison with previous studies exemplifies the benefit of larger samples for identifying trends in skin cancer lipids [42, 43], thus an increased sample size can improve confidence in findings of differential expression metabolomic analysis and can reduce the amount of falsely detected signals. Additionally, this study was performed on *ex-vivo* samples extracted by standard excision biopsy which might produce different results than an *in-vivo* analysis. Lastly, patient inclusion criteria were not very strict, potentially overlooking patient lifestyle information that could provide useful information such as levels of sun exposure.

Several studies are planned for the next steps. First, we aim to conduct validation experiments to confirm the reliability of the identified differentially expressed metabolites. Second, we plan to perform experiments with multiple samples per lesion to assess the feasibility of constructing spatial metabolomic maps, to evaluate intra-lesion heterogeneity [13, 44], and to estimate the reproducibility of the e-biopsy sampling approach.

The early-stage results of the e-biopsy sampling technique show promise of its potential development as a handheld device that limits the need for tissue resection during biopsy. Its ability to be used as a standalone device provides advantages over tools such as the iKnife [26, 27] and MasSpec Pen [28, 29] in primary care settings. In contrast to other needle biopsy techniques such as fine-needle aspiration and core needle biopsy, needle size is not a limiting factor [45, 46], and a more invasive larger needle diameter is not needed for greater accuracy [47, 48]. Conversely, e-biopsy can provide valuable site-specific information by its ability to sample areas larger than the needle diameter [16] which provides opportunities for greater understanding of tumor complexities and mapping of tumor heterogeneity [15]. Additionally, in pathology lab settings, e-biopsy could streamline workflows by integrating molecular profiling with AI-driven diagnostic tools, offering enriched diagnostic reports before traditional staining and evaluation. This innovation has the potential to reduce pathology workloads while enhancing diagnostic accuracy and facilitating precision medicine.

The e-biopsy methodology, developed for skin cancer diagnostics, has potential for application to other cancers and tissue types. Its minimally invasive nature and ability to sample molecular content through pulsed electric fields (PEF) make it particularly well-suited for both accessible tumors, such as breast and head-and-neck cancers, and deep-seated malignancies, such as pancreatic, colon or liver cancers, when paired with endoscopic or image-guided approaches. Furthermore, e-biopsy offers the unique advantage of capturing spatial molecular profiles from multiple locations within a tumor, enabling the assessment of intra-tumoral heterogeneity [13, 44]—a critical factor for understanding tumor biology and treatment response.

Beyond oncology, the platform may be adapted to study chronic conditions in other tissues, such as liver fibrosis or neurodegenerative diseases, through its capacity to analyze proteins, lipids, metabolites, and RNA. Compared to traditional excision biopsies, e-biopsy reduces tissue damage and potentially reducing patient discomfort, while preserving the integrity of sampled material for multi-omics analyses. Future studies will explore its adaptability to various tissue microenvironments, optimize protocols for new applications, and assess its integration with existing diagnostic and therapeutic workflows, offering a promising pathway for advancing precision medicine across a range of diseases.

## Conclusion

This study contributes to the understanding of cSCC and BCC molecular mechanisms. We report high-throughput metabolomics profiles of cSCC, BCC and healthy skin, together with a comparative analysis between these tissue types. A total of 2325 metabolites were identified, among them 301 with high confidence. In that group 34 metabolites were found to be differentially expressed. The differentially expressed metabolites were from several subclasses (e.g. amino acids, peptides, and analogues, and carbohydrates and carbohydrate conjugates). Measurement details can be found in the supplementary material (Tables S1–S4). Overall trends in the data indicate a greater number of amino acids, peptides, and analogues with higher expression in both cSCC and in BCC compared to healthy skin tissue. This study demonstrates the opportunity in high-throughput metabolomic profiling of e-biopsy-gathered samples and its potential for enhancing skin cancer diagnostic methods.

## Supplementary Information

Below is the link to the electronic supplementary material.

Supplementary file1 (JPG 373 KB)

Supplementary file2 (JPG 380 KB)

Supplementary file3 (JPG 375 KB)

Supplementary file4 (JPG 504 KB)

Supplementary file5 (JPG 382 KB)

Supplementary file6 (JPG 375 KB)

Supplementary file7 (JPG 391 KB)

Supplementary file8 (JPG 438 KB)

Supplementary file9 (JPG 364 KB)

Supplementary file10 (JPG 375 KB)

Supplementary file11 (JPG 372 KB)

Supplementary file12 (JPG 361 KB)

Supplementary file13 (XLSX 325 KB)

Supplementary file14 (XLSX 99 KB)

Supplementary file15 (XLSX 315 KB)

Supplementary file16 (XLSX 193 KB)

Supplementary file17 (XLSX 11 KB)

Supplementary file18 (XLSX 10 KB)

Supplementary file19 (XLSX 10 KB)

Supplementary file20 (DOCX 36 KB)

Supplementary file21 (DOCX 13 KB)

## Data Availability

All the data that supports the findings of this study are available in the supplementary materials.

## Abbreviations

BCC: Basal cell carcinoma
cSCC: Cutaneous squamous cell carcinoma
e-Biopsy: Electroporation-based biopsy
UPLC-MS-MS: Ultra performance liquid chromatography and tandem mass spectrometry
