# Supplementary figures and images for: High-Throughput Metabolomic Profiling of Skin Lesions: Comparative Study of Cutaneous Squamous Cell Carcinoma, Basal Cell Carcinoma, and Normal Skin Via e-Biopsy Sampling
Source: Cell Mol Bioeng. 2025 Apr 3;18(2):185–95. doi: 10.1007/s12195-025-00846-1 (PMC12018666; doi:10.1007/s12195-025-00846-1)

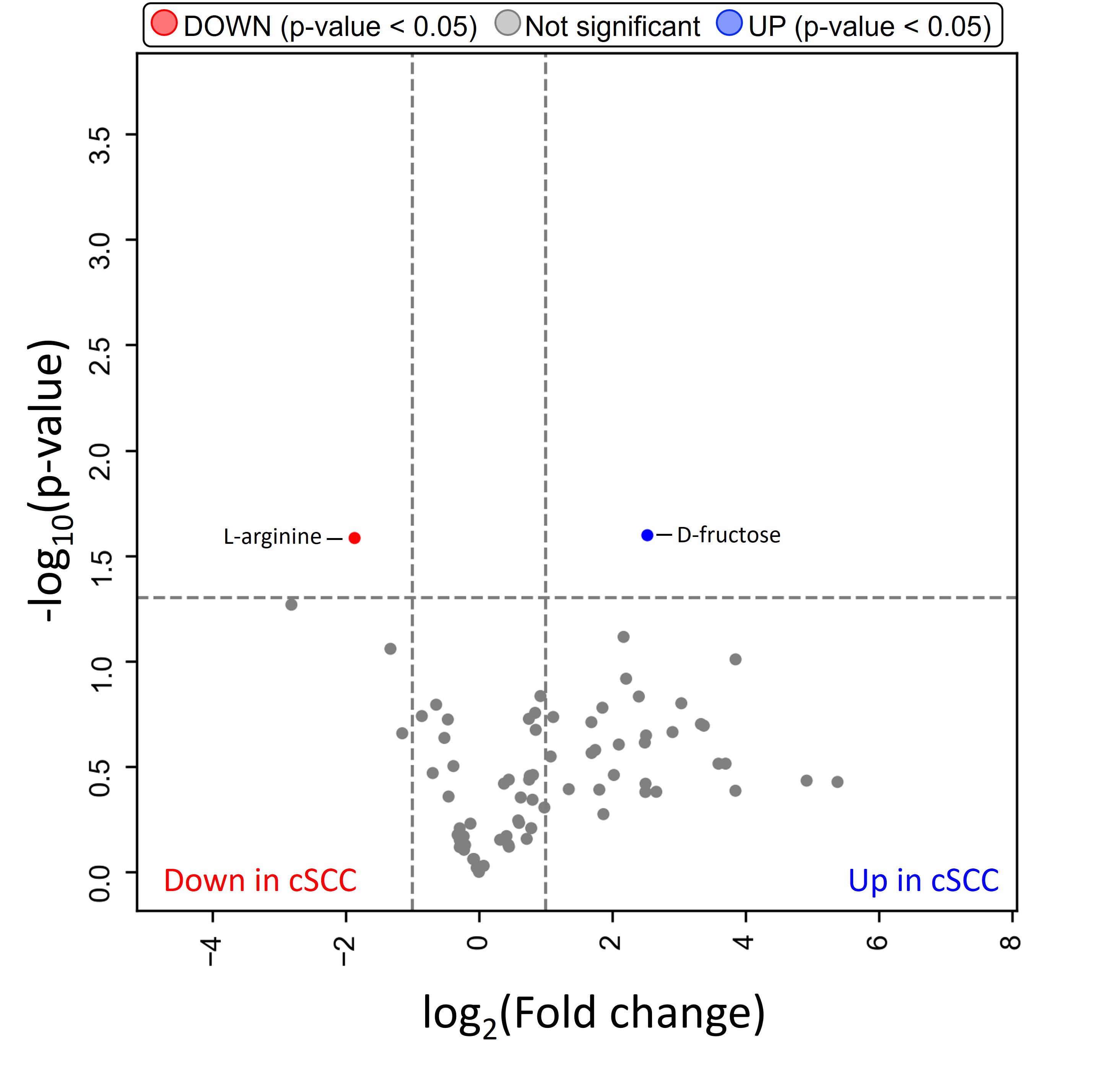

Supplement: Supplementary file 1 — Supplementary file1 (JPG 373 KB) [file 12195_2025_846_MOESM1_ESM.jpg]

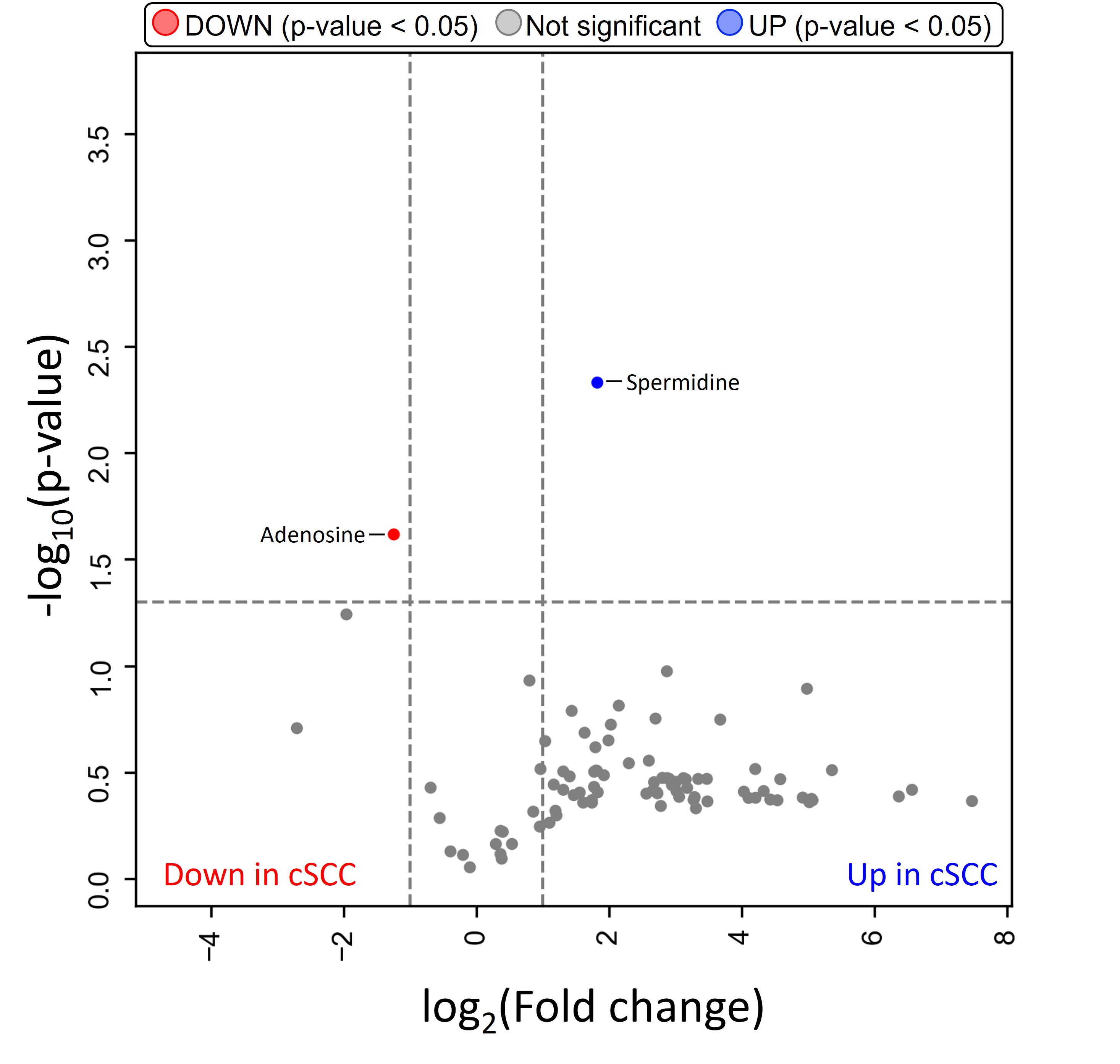

Supplement: Supplementary file 2 — Supplementary file2 (JPG 380 KB) [file 12195_2025_846_MOESM2_ESM.jpg]

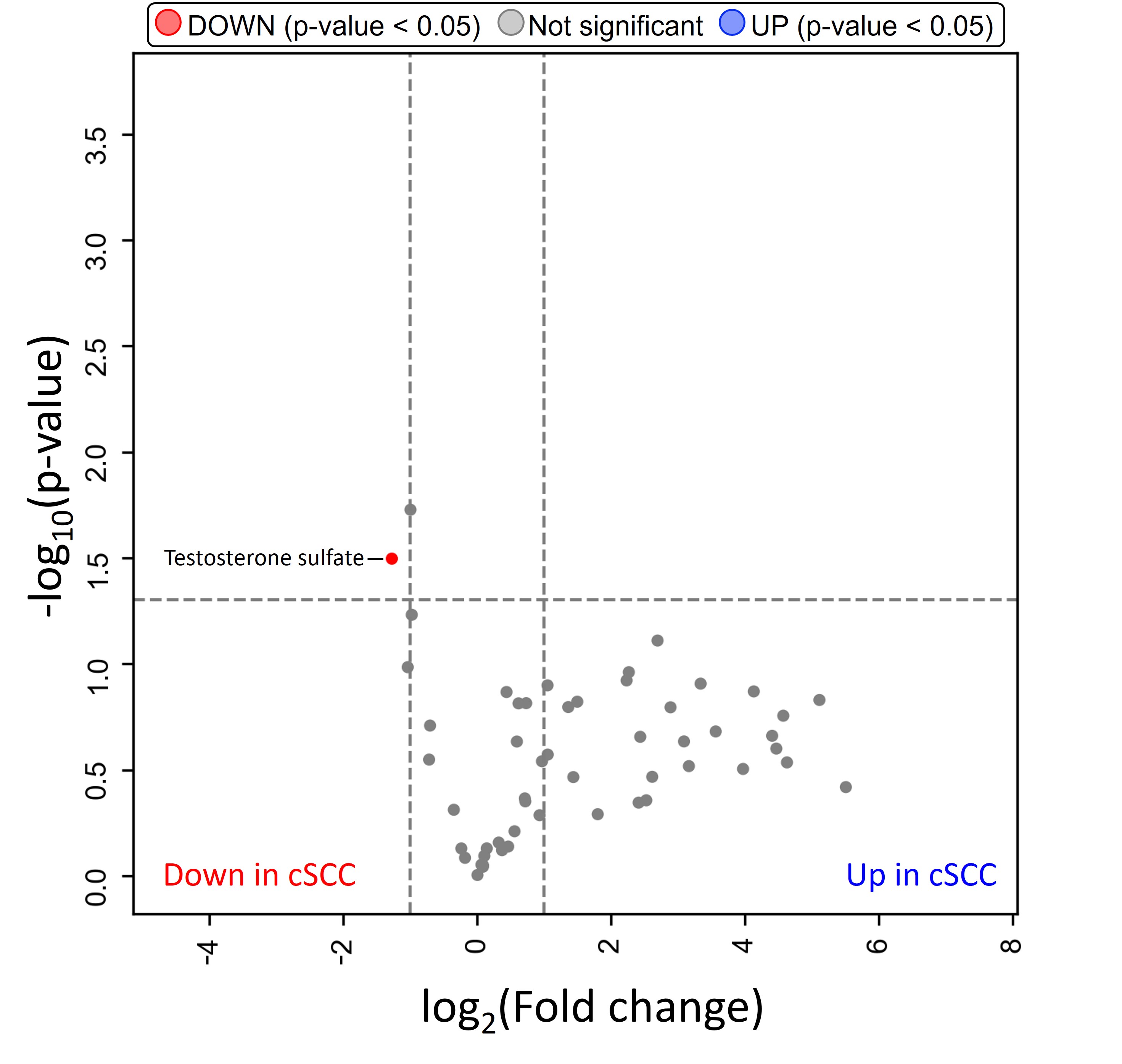

Supplement: Supplementary file 3 — Supplementary file3 (JPG 375 KB) [file 12195_2025_846_MOESM3_ESM.jpg]

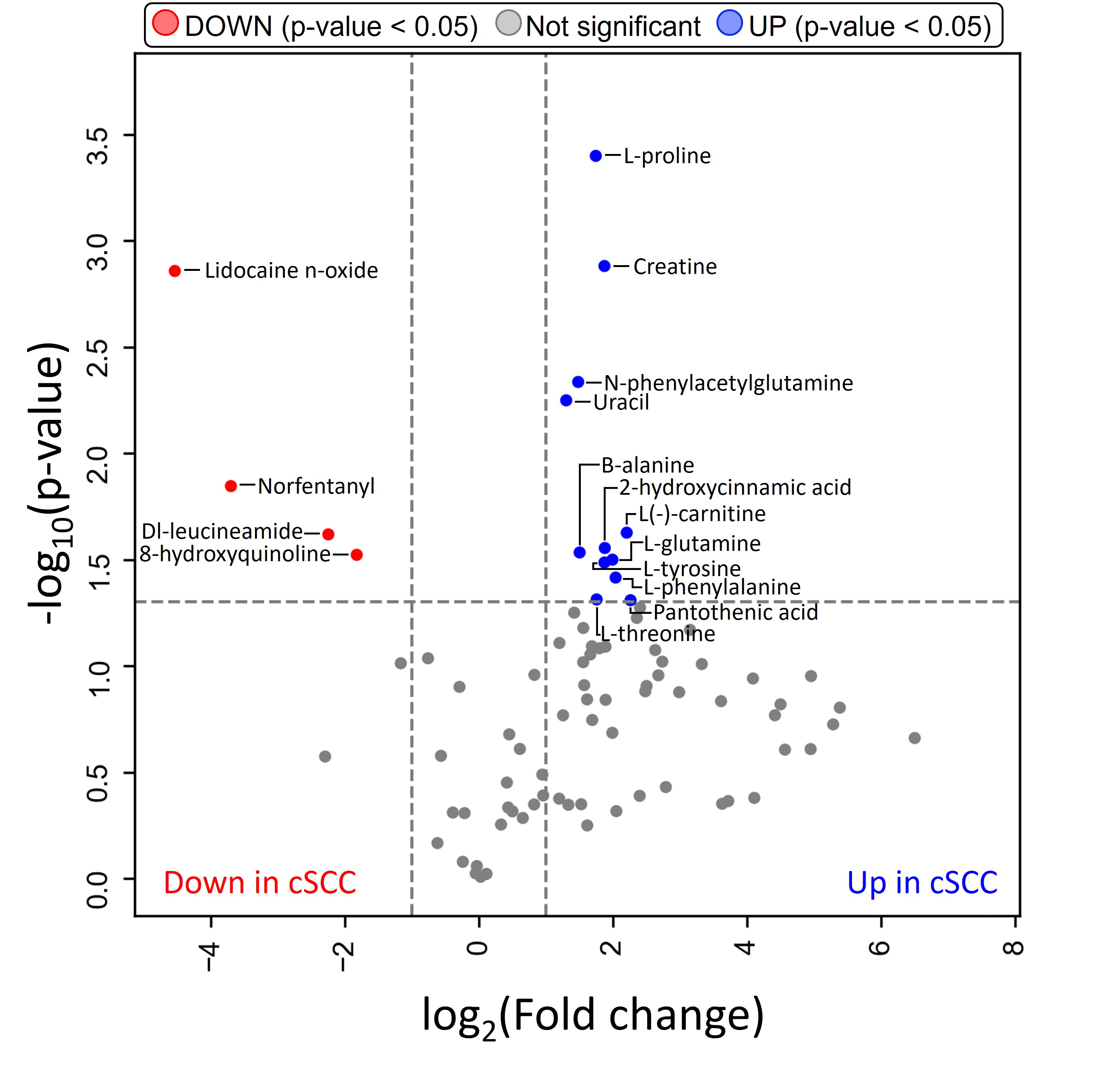

Supplement: Supplementary file 4 — Supplementary file4 (JPG 504 KB) [file 12195_2025_846_MOESM4_ESM.jpg]

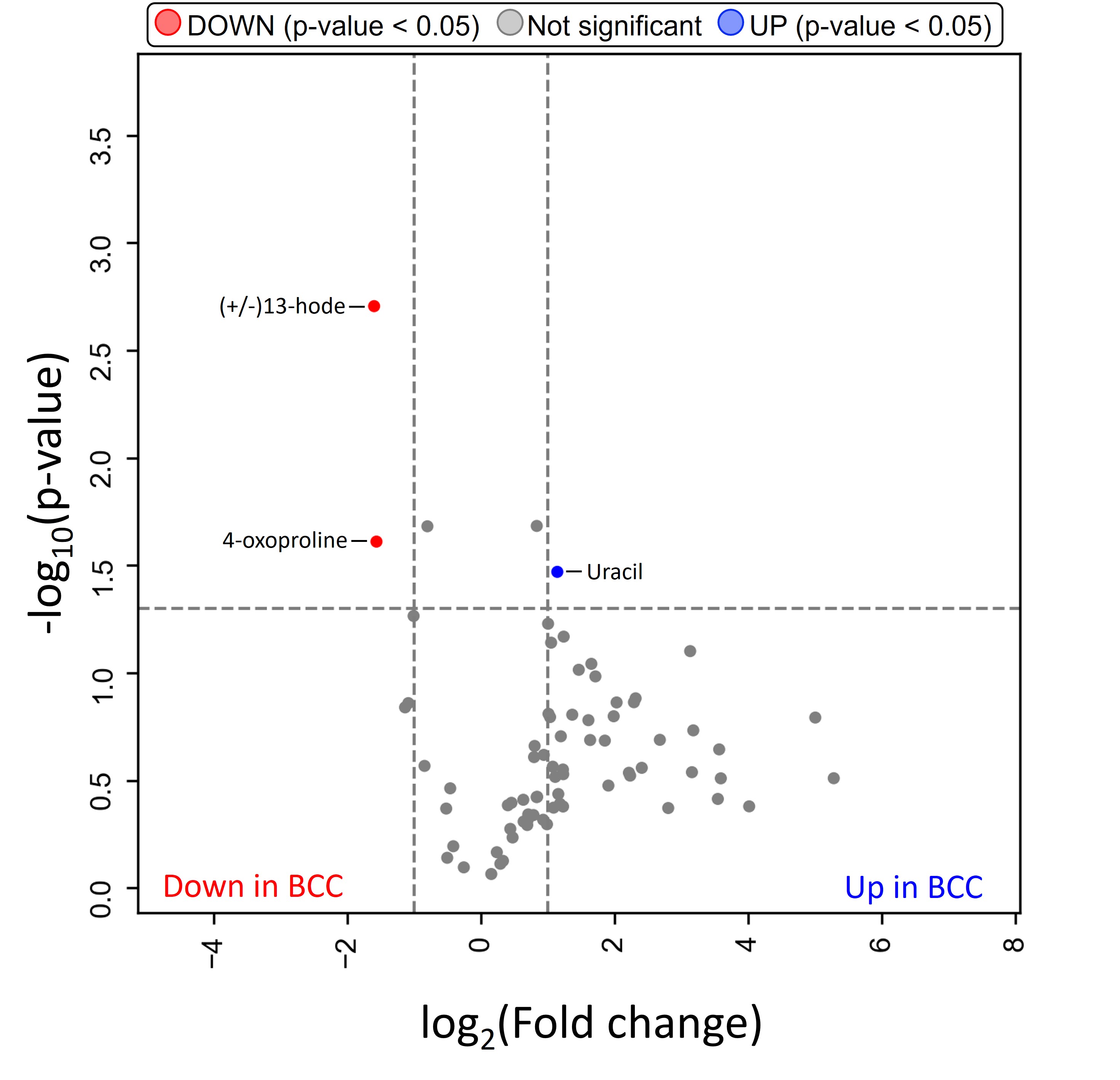

Supplement: Supplementary file 5 — Supplementary file5 (JPG 382 KB) [file 12195_2025_846_MOESM5_ESM.jpg]

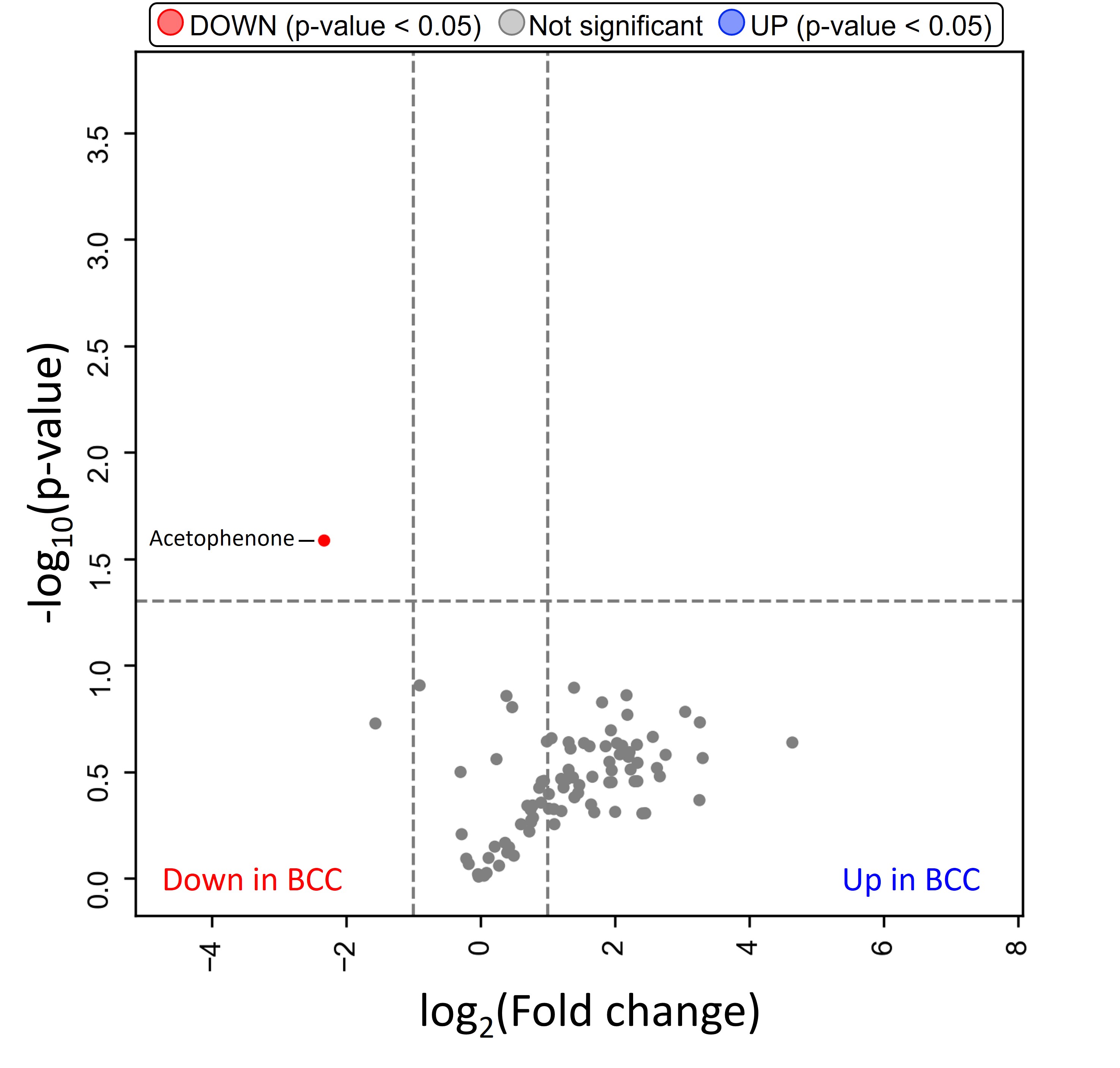

Supplement: Supplementary file 6 — Supplementary file6 (JPG 375 KB) [file 12195_2025_846_MOESM6_ESM.jpg]

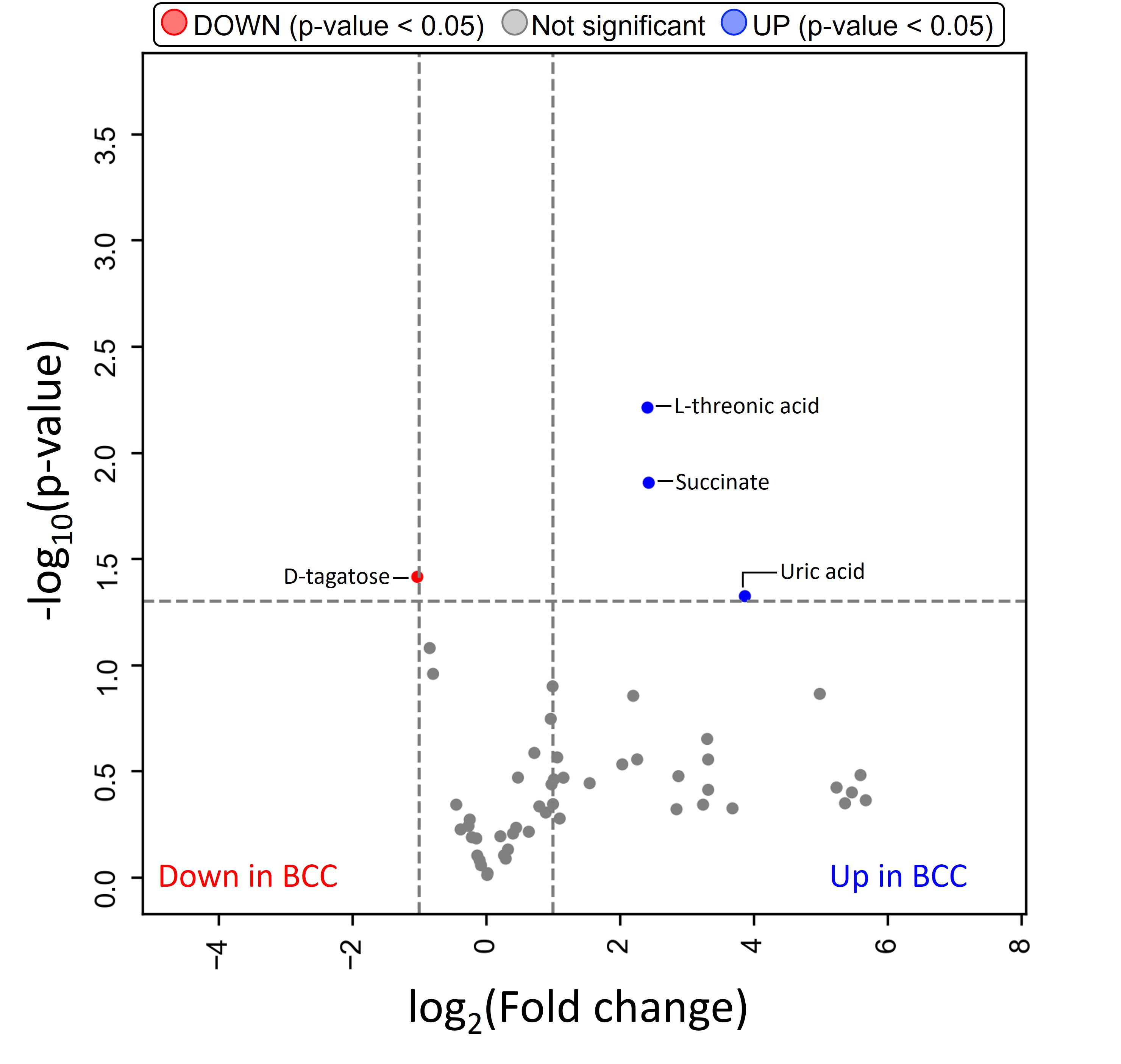

Supplement: Supplementary file 7 — Supplementary file7 (JPG 391 KB) [file 12195_2025_846_MOESM7_ESM.jpg]

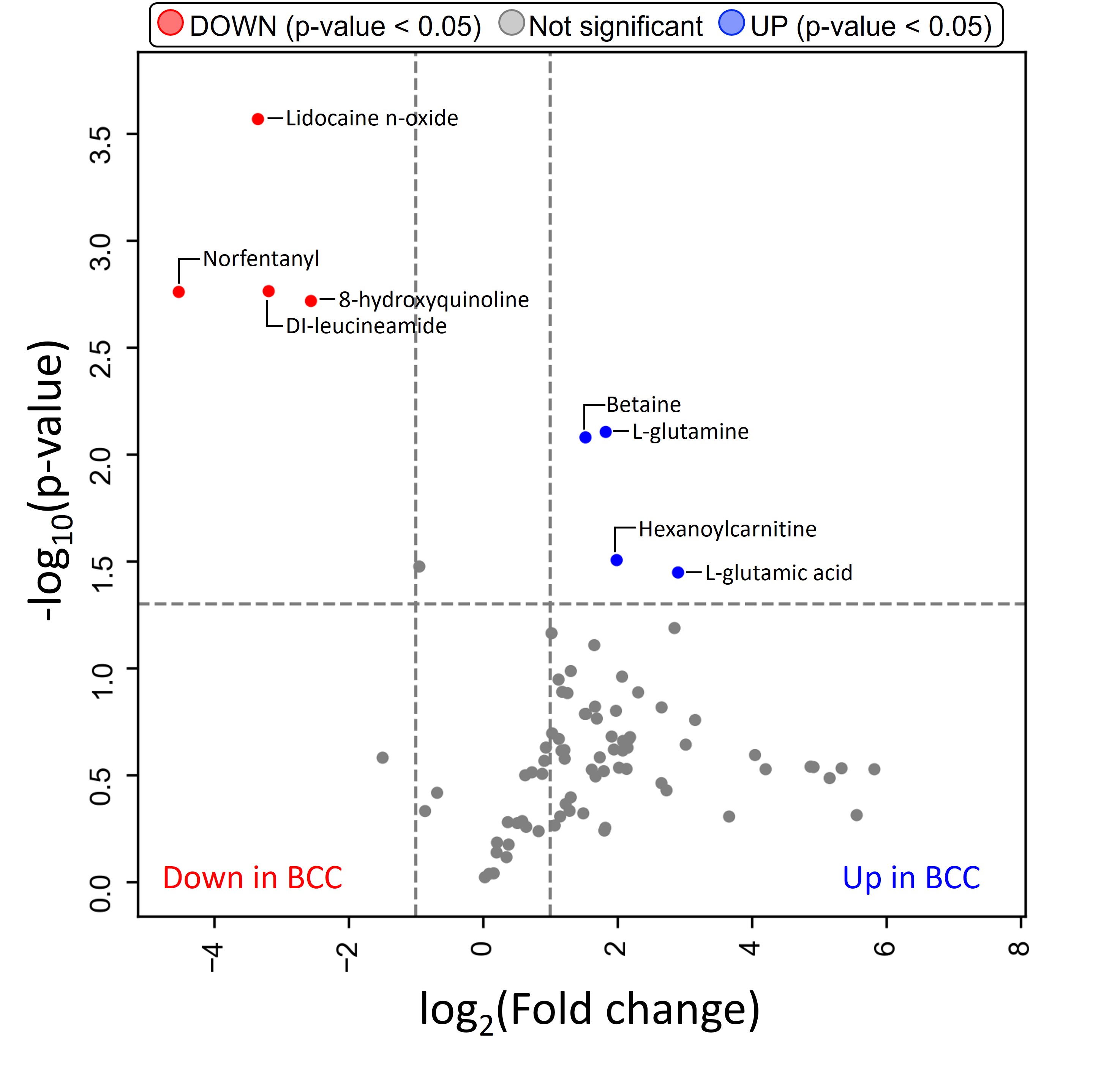

Supplement: Supplementary file 8 — Supplementary file8 (JPG 438 KB) [file 12195_2025_846_MOESM8_ESM.jpg]

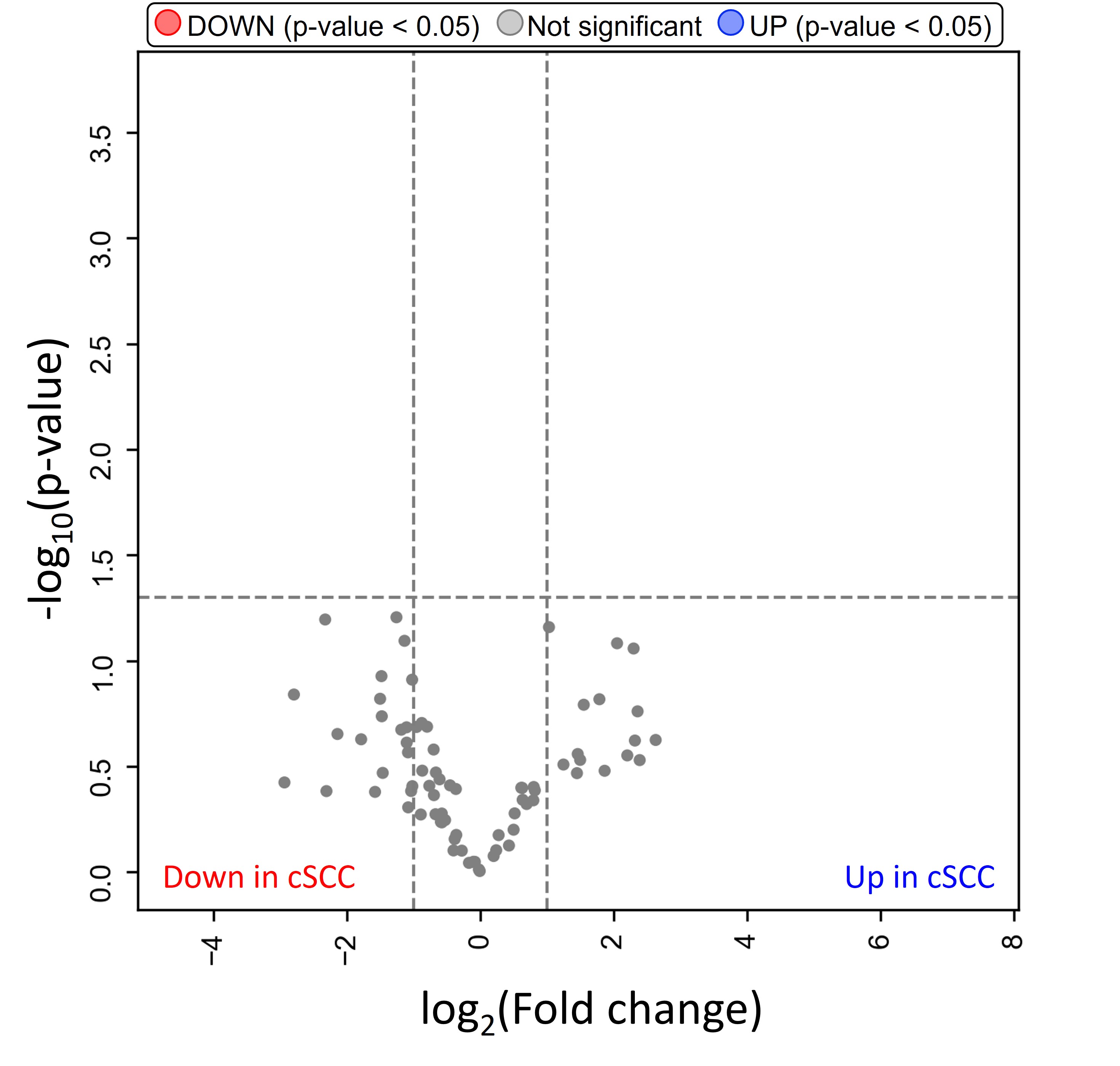

Supplement: Supplementary file 9 — Supplementary file9 (JPG 364 KB) [file 12195_2025_846_MOESM9_ESM.jpg]

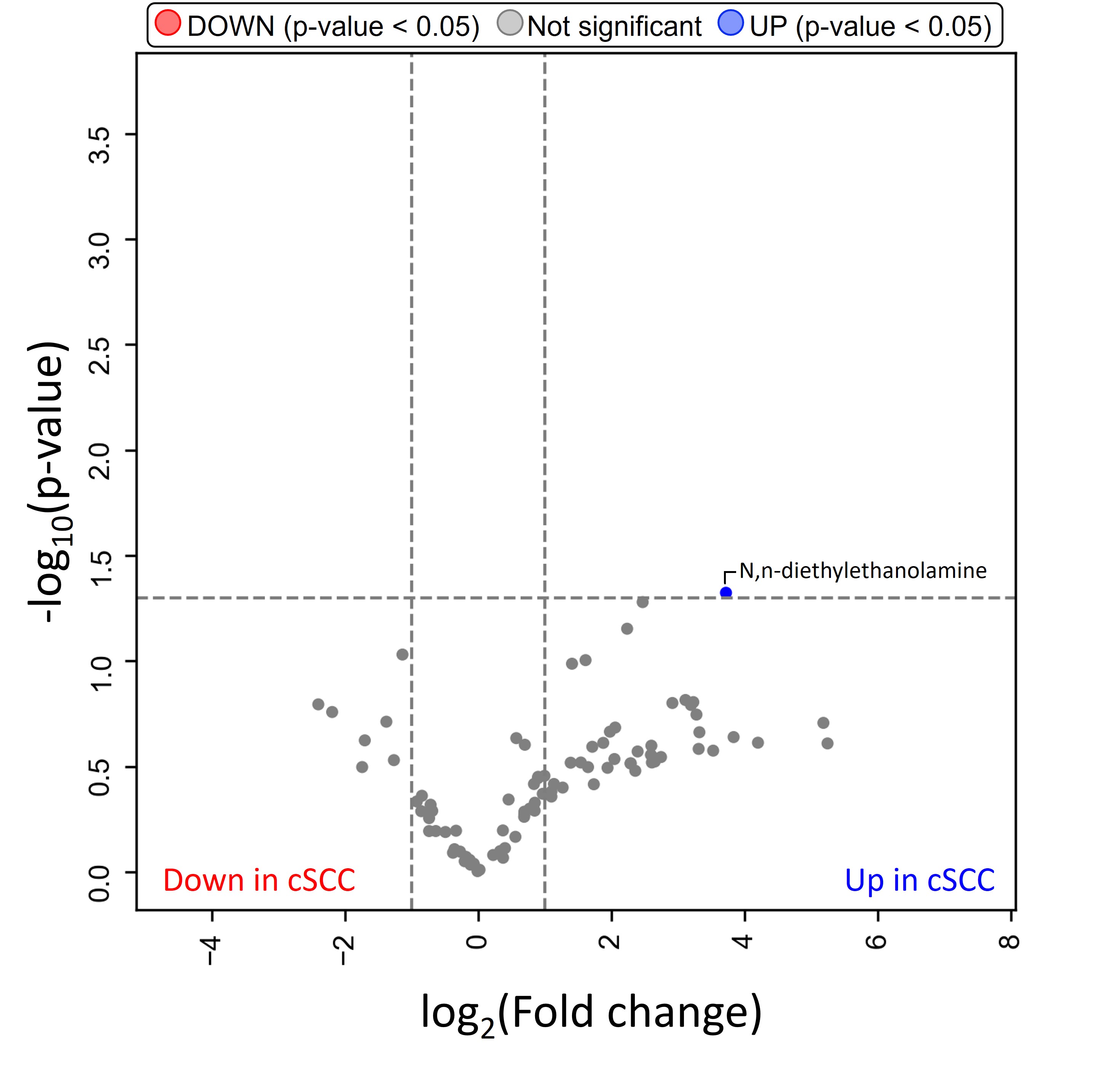

Supplement: Supplementary file 10 — Supplementary file10 (JPG 375 KB) [file 12195_2025_846_MOESM10_ESM.jpg]

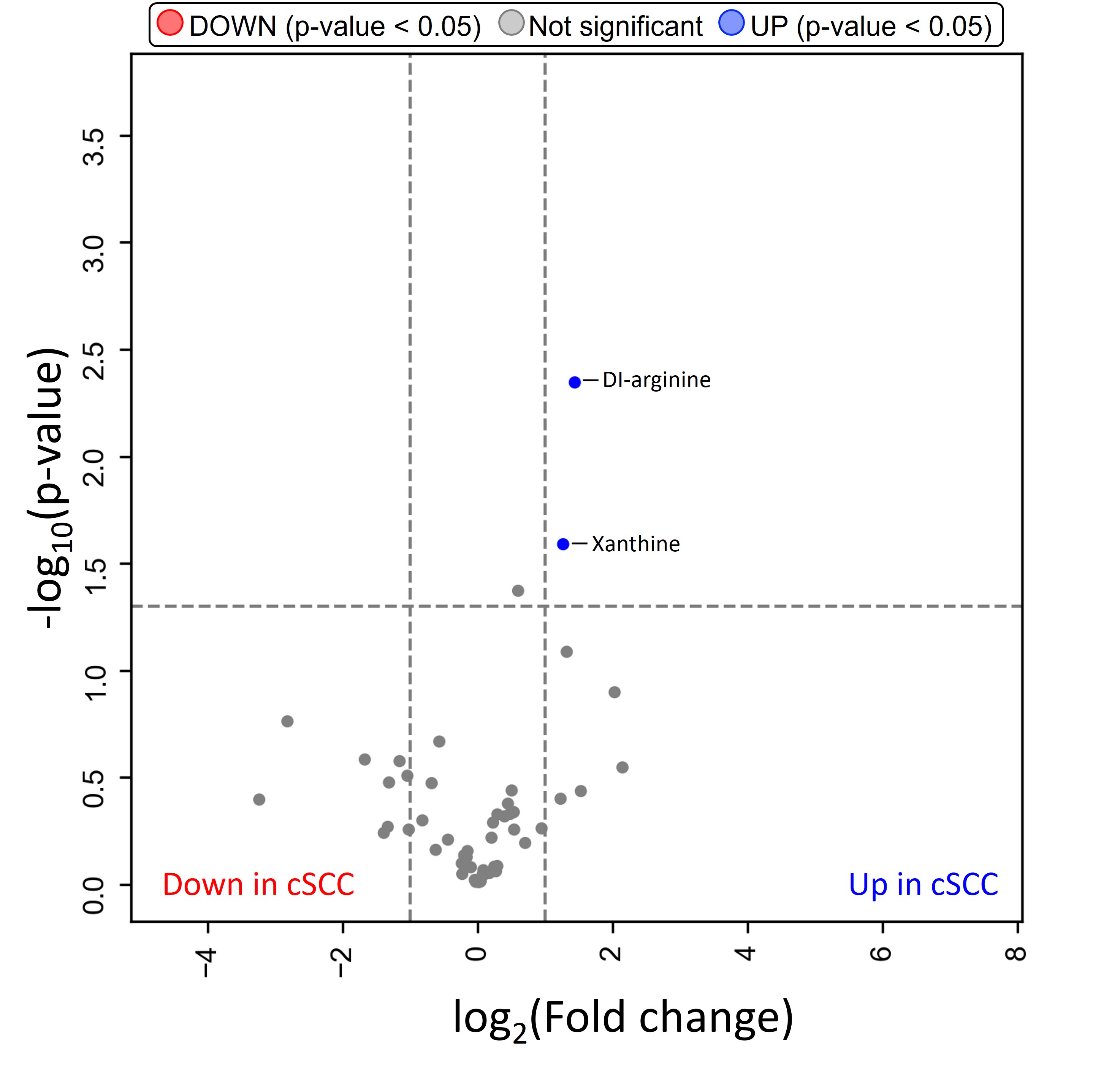

Supplement: Supplementary file 11 — Supplementary file11 (JPG 372 KB) [file 12195_2025_846_MOESM11_ESM.jpg]

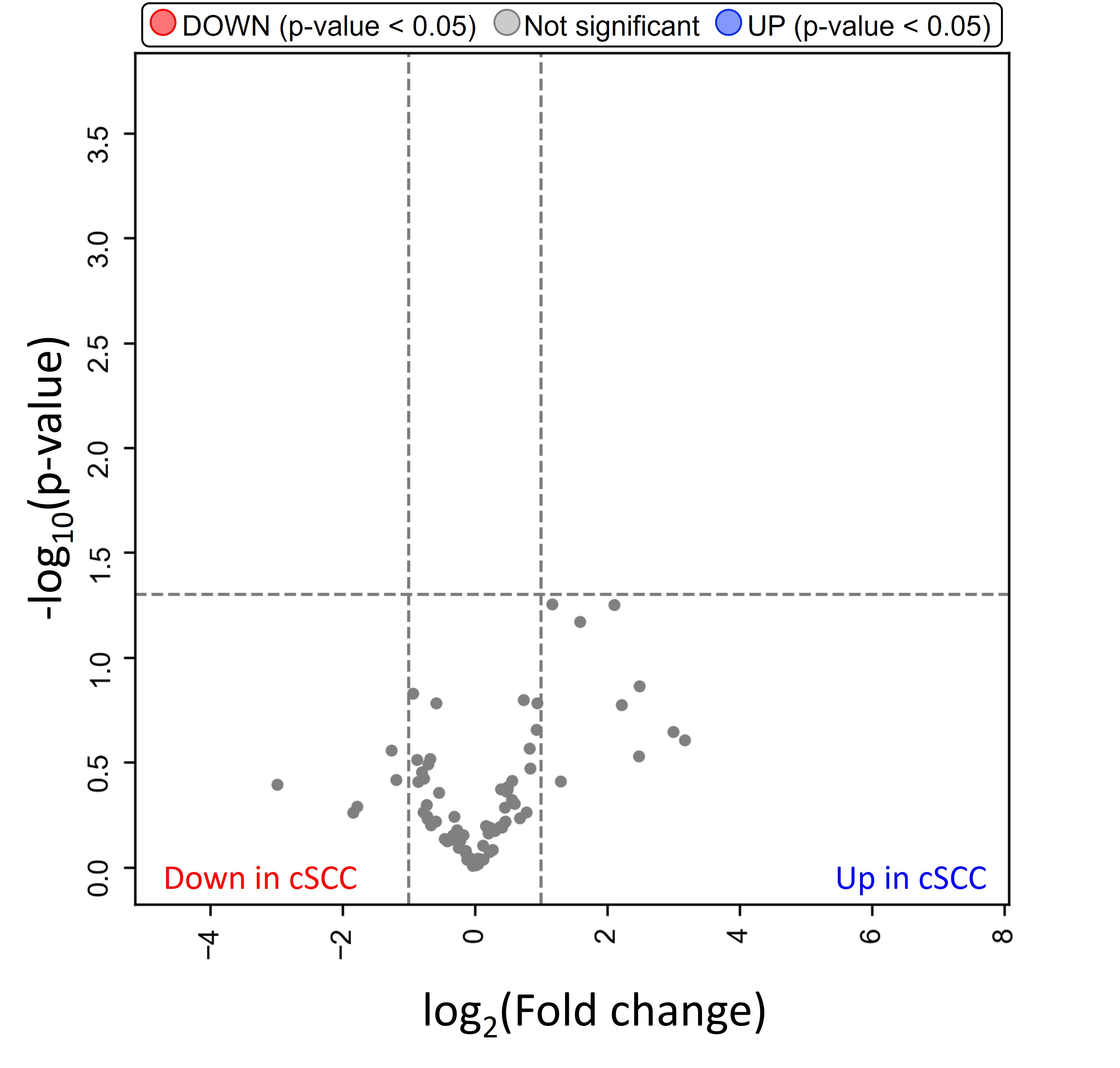

Supplement: Supplementary file 12 — Supplementary file12 (JPG 361 KB) [file 12195_2025_846_MOESM12_ESM.jpg]
